# Diagnostic procedure for idiopathic eosinophilic pleural effusion: a single-center experience

**DOI:** 10.1186/s12890-020-1108-z

**Published:** 2020-04-03

**Authors:** Weizhan Luo, Yunxiang Zeng, Panxiao Shen, Jianxing He, Jinlin Wang

**Affiliations:** 1 Department of Respiratory Disease, The State Key Laboratory of Respiratory Disease, China Clinical Research Centre for Respiratory Disease, Guangzhou Institute of Respiratory Health, First Affiliated Hospital of Guangzhou Medical University, 151 Yanjiang Road, Guangzhou, 510120 Guangdong Province China; 2 Department of Cardiothoracic Surgery, The State Key Laboratory of Respiratory Disease, China Clinical Research Centre for Respiratory Disease, Guangzhou Institute of Respiratory Health, First Affiliated Hospital of Guangzhou Medical University, 151 Yanjiang Road, Guangzhou, 510120 Guangdong Province China

**Keywords:** Eosinophilic pleural effusion, Idiopathic, Diagnosis, Glucocorticoid, Diagnostic procedure

## Abstract

**Background:**

Eosinophilic pleural effusion (EPE) is attributed to several well-recognised causes. However, some patients remain idiopathic, even after thorough clinical work-up. The present study aimed to better characterize idiopathic EPE (IEPE) and to outline the diagnostic procedure for this disease.

**Methods:**

Complete clinical data of 11 consecutive patients with IEPE were prospectively collected and analysed. Preliminary diagnostic procedure of IEPE in our hospital was performed.

**Results:**

All the 11 patients had respiratory symptoms and unilateral pleural effusion (PE) occurred in 4 patients. The mean percentage of eosinophils in PE was 22.4% (range, 12.4–50.5%). Lactate dehydrogenase, adenosine deaminase, proteins and carcinoembryonic antigen in PE were 246.0 U/L (range, 89.8–421.9 U/L), 13.8 U/L (range, 1.8–24.0 U/L), 42.6 g/dl (range, 32.8–52.6 g/dl) and 2.17 mg/mL (range, 0.46–4.31 mg/mL), respectively. Parasite-specific IgG antibody in blood and parasite eggs in stool were both negative. No evidence of tuberculosis or malignancy was observed in pleural biopsy. Symptoms and abnormal pulmonary imaging were eliminated after glucocorticoid use.

**Conclusions:**

IEPE is a diagnosis of exclusion. Patients with EPE without a clear cause should be asked to provided complete medical, surgical and drug-related histories. A thorough work-up is essential. Moreover, we recommend follow-up after the use of glucocorticoid until effusion resolves.

**Trial registration:**

GYFYY. Registration No: GYFYY20150901221. Registered time: 1 September 2015. Date of enrolment of the first participant to the trial: 22 January 2016.

## Background

Pleural effusion (PE) is a very common clinical and radiological finding in respiratory medicine [1, 2]. The diagnosis of PE includes both non-invasive and invasive approaches. Thoracic ultrasound, thoracic computed tomography (CT) scan and positron emission tomography with ^18^F-fluorodeoxyglucose (FDG-PET CT) chemical confirm its presence [3]. Pleuroscopy, thoracentesis, laboratory tests, and cytological analysis, provide further information about the aetiology of the disease and thus, are also essential [2, 3]. Eosinophilic pleural effusion (EPE) is defined as PE that demonstrates at least 10% eosinophils within a white cell differential count [4, 5], accounting for 5 to 16% of the total exudative PEs. There are several aetiological factors for EPE including trauma, infectious diseases, malignant tumours, asbestos exposure and several medications [4–8]. However, of the patients with EPE, approximately 14–25% are diagnosed as idiopathic, even after thorough clinical work-up. In EPE cases where a specific aetiology remains undetermined, the diagnosis is idiopathic EPE (IEPE). IEPE is likely to benefit from the use of glucocorticoids [7–11].

The reported prevalence of IEPE is inconsistent. In Adelman’s study [4], 35% of patients with EPE had no apparent causes. In contrast, another study reported only 8.5% [11]. A 2012 meta-analysis and systematic review [7] concluded that the two most common causes of EPE are malignancy (26%) and IEPE (25%). Compared with other forms of EPE, EPE is more likely to be idiopathic [7].

Although IEPE has been regarded as an important cause of EPE, few prospective studies are available [12–16]. The clinical characteristics and diagnostic approach for IEPE have remained unclear to physicians. Delayed diagnosis and/or misdiagnosis probably may lead to significant morbidity and even mortality. In order to better characterize IEPE and to outline its diagnostic procedure, comprehensive clinical data of 11 consecutive patients with EPE was collected and analysed in the prospective study. Importantly, a preliminary diagnostic procedure of IEPE was introduced.

## Methods

### Patients

Five hundred and 56 consecutive patients with PE were admitted to the First Affiliated Hospital of Guangzhou Medical University due to respiratory symptoms between January 2016 and January 2018. Four hundred and 82 patients were scanned using high-resolution chest CT (HRCT) and had PE or pleural pulmonary involvement, but those with PE showing eosinophils of less than 10% were excluded from this study. Past medical, surgical, traumatic infectious and drug-related histories were obtained from a total of 74 patients with EPE. These patients also received extensive work-up to identify a definite aetiology for their EPE. After excluding EPE cases with known aetiological factors, the complete clinical data of 11 patients with IEPE were prospectively collected and analysed. Informed consent was obtained from all patients for the use of identified personal data extracted from their medical records for research purposes only. The study was approved by the Ethics Committee of the First Affiliated Hospital of Guangzhou Medical University.

### Exclusive diagnosis

Before further laboratory testing and imaging examination for EPE, it is necessary to review a patient’s past medical and surgical history to identify any potential primary treatable cause/s. Additionally, it is important to review of any drug intake, occupational and infectious disease exposure and comorbid conditions to rule out the common causes of EPE. The common aetiology of EPE including malignant PE (MPE), tubercular PE (TPE), parapneumonic PE (PPE) and pleural parasitic infestation (PPI) were excluded by laboratory tests.

### Pleural biopsy

Pleural samples were acquired by combined ultrasound-guided cutting needle biopsy and standard pleural biopsy [17].

### Laboratory measurements

Biochemical analysis (total protein, lactate dehydrogenase), bacterial, fungal and mycobacterial culture, Gram stain, and cytological examinations were performed for all PE samples. Meanwhile, total protein and lactate dehydrogenase in the serum were measured by standard methods. Pleural BNP levels were determined in an autoanalyser using the commercially available enzyme immunoassay kit (Roche) following the manufacturer’s instructions.

The differential count of the nucleated cells was done after cytocentrifuging (2500 r/min for 7 min) and HE stain was done manually for the pleural liquid. EPE was defined as pleural effusion with ≥10% eosinophils.

### Diagnosis of pleural parasitic infestation (PPI)

The diagnostic approach for PPI was as previously described by the authors [18]. The enzyme-linked immunosorbent assay (ELISA) test for parasite-specific IgG antibodies (Guangzhou Yikang Biotechnology Co. Ltd.) was performed on serum from all patients. The parasite-specific IgG antibodies included the IgG antibodies of *Taenia solium, Paragonimus westermani,* and *Spirometra spp., Clonorchis sinensis, Toxoplasma gondii and Echinococcus granulosus*. Stool examinations for the detection of parasite eggs were performed in all patients.

## Results

### Characteristics of patients with IEPE

In total, complete clinical data sets of 11 patients with IEPE were collected and analysed in this study. The clinical characteristics of 11 cases are summarized in Table 1. Three were 5 men and 6 women, with a median age of 49.8 years (range, 30–67 years). All cases had respiratory symptoms including shortness of breath (*n* = 10), cough (*n* = 3), chest pain (*n* = 3), fever (*n* = 3) and excessive sputum (*n* = 1). The duration of these symptoms ranged from 15 days to more than 2.5 months. Pulmonary physical examination revealed remarkably decreased breath sounds with dullness to percussion on the lateral or bilateral chests, without other significantly positive signs. Among the 11 patients, 2 presented with left PE, 2 others presented with right PE, and the remaining 7 had bilateral PE. The diagnosis was similar to PPE in 3 cases. Five cases were initially misdiagnosed with TPE. A patient was considered as having MPE, and another patient was misdiagnosed with chronic heart failure (CHF).

**Table 1:** Demographic characteristics of 11 patients with IEPE

No	Chief complaint (duration)	Misdiagnosis	Side of PE	Pathology of pleura biopsy	Treatment	Follow-up (mth)	Other organ involvement
1	Fever, shortness of breath (20 d)	TPE	Bilateral	Eosinophilic infiltration	Glucocorticoid	9	lung
2	shortness of breath (2 mth)	N	Right	Eosinophilic infiltration	Glucocorticoid	15	N
3	Chest pain, shortness of breath (1 mth)	PPE	Bilateral	Lymphocytic infiltration	Glucocorticoid	12	Lung pericardium
4	Cough, shortness of breath (1 mth)	TPE	Bilateral	Noncaseating granulomas	Glucocorticoid	8	N
5	Shortness of breath (2.5 mth)	TPE	Bilateral	Lymphocytes infiltration	Glucocorticoid	16	N
6	Chest pain, shortness of breath (1 mth)	MPE	Left	Eosinophilic infiltration	Glucocorticoid	11	Lung
7	Cough, excessive sputum, shortness of breath (1 mth)	CHF	Bilateral	Eosinophilic infiltration	Glucocorticoid	10	N
8	Fever, shortness of breath (1 mth)	TPE	Left	Lymphocytic infiltration	Glucocorticoid	12	N
9	Cough, chest pain, shortness of breath (2 mth)	PPE	Bilateral	Eosiniphilic & lymphocytic infiltration	Glucocorticoid	11	Pericardium
10	Fever, cough (15 d)	PPE	Bilateral	Granulocytic & lymphocytic infiltration	Glucocorticoid	10	Lung
11	Shortness of breath (2 m)	TPE	Right	Eosinophilic infiltration	Glucocorticoid	11	N

*Abbreviations: IEPE* idiopathic eosinophilic pleural effusion, *F* female, *d* day, *TPE* tuberculosis pleural effusion, *M* male, *mth* month, *N* none, *PPE* parapneumonic pleural effusion, *MPE* malignant pleural effusion, *CHF* chronic heart failure

### Laboratory tests for peripheral blood cell (PBC) and serological examination

PBC analysis was conducted in all patients (Table 2). Leukocytosis of peripheral blood (> 10 × 10^9^/L) was observed in 4 cases (case 2, 3, 7 and 11), and eosinophilia (> 0.5 × 10^9^/L) was seen in 5 cases (case2, 3, 5, 7 and 11). No specific findings were observed in blood tests, including liver function, thyroid function, C-reactive protein, erythrocyte sedimentation rate, and interferon-γ release assays (IGRAs), carcinoembryonic antigen (CEA) and brain natriuretic peptide (BNP). Antinuclear antibody, rheumatoid factor antibody, proteinase 3, myeloperoxidase and anticyclic citrullinated peptide antibody were not detected. Sputum smears and cultures for fungi, acid-fast bacilli and other bacteria were also negative. In addition, the test for parasite-specific IgG antibody was negative, and parasite eggs were not found in any stool samples.

**Table 2:** Blood examinations of 11 patients with IEPE

No	WBC (×10^9^/L)	Eos (×10^9^/L)	CEA (ng/mL)	LDH (U/L)	BNP (pg/mL)	ANA (U/mL)	PR3 (U/mL)	MPO (U/mL)	ESR (mm/h)	IGRAs	IgE (U/mL)	Parasite-specific IgG antibodies	Parasite eggs from stool
1	3.21	0.49	2.86	130	100	4.45	3.47	2.15	100	N	–	N	N
2	13.57	2.16	1.80	178	17.43	5.66	4.87	4.80	35	N	223	N	N
3	11.2	0.6	0.67	164	248.50	5.02	0.47	1.29	66	N	339	N	N
4	4.8	0.36	1.38	146	30.41	2.68	2.14	4.64	25	N	–	N	N
5	3.93	0.71	2.17	182	44.56	11.57	1.95	3.79	43	N	243	N	N
6	4.78	0.49	3.23	201	321.32	3.50	2.43	4.23	47	N	–	N	N
7	11.3	0.64	2.10	143	453.45	4.38	2.56	2.46	56	N	–	N	N
8	4.56	0.35	4.56	203	487.23	3.56	2.45	2.67	46	N	115	N	N
9	8.6	0.42	3.56	189	123.2	4.32	3.43	2.87	54	N	231	N	N
10	8.65	0.34	2.89	212	212.67	5.43	4.23	3.45	34	N	–	N	N
11	10.38	0.84	3.56	156	325	4.21	1.24	2.43	46	N	165	N	N

*Abbreviations: IEPE* idiopathic eosinophilic pleural effusion, *WBC* white blood cell, *Eos* eosinophils, *CEA* carcinoembryonic antigen, *LDH* lactate dehydrogenase, *BNP* brain natriuretic peptide, *ANA* antinuclear antibodies, *PR3* proteinase 3, *MPO* myeloperoxidase, *IGRAs* interferon-γ release assays, *N* negative

### Invasive work-up

**Fig. 1:**
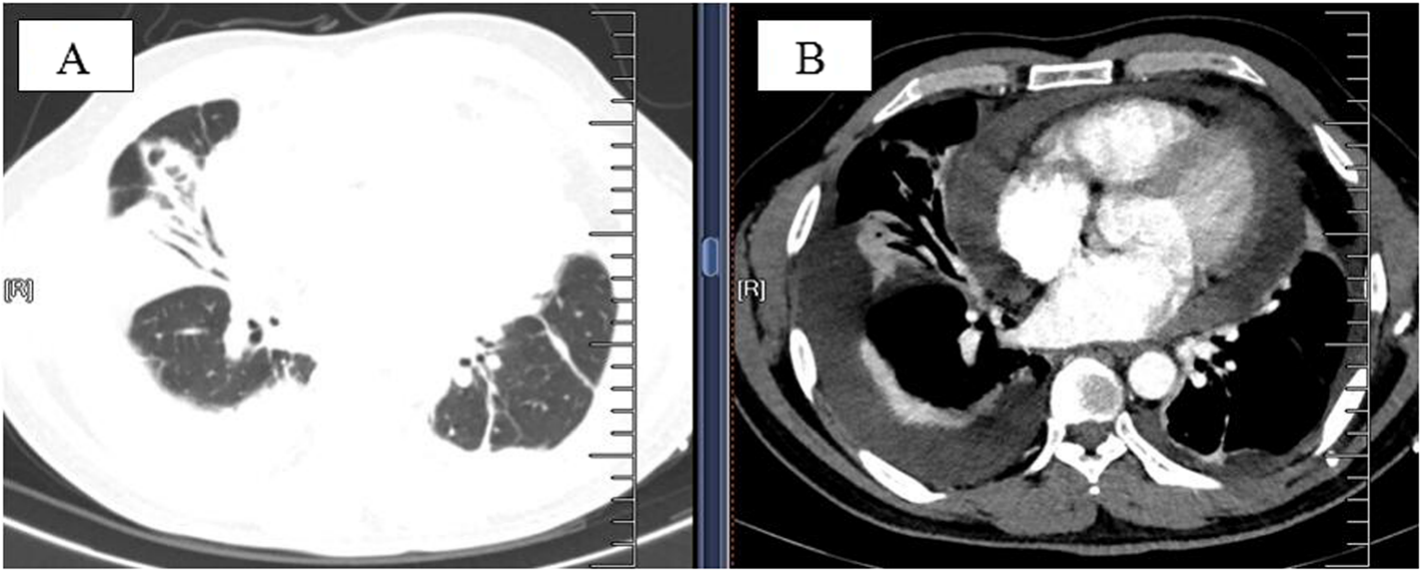
**a** 44-year-old male patient with IEPE (case 3). Chest CT **a, b** scans showed bilateral PE and consolidation in the lower right lung, pericardial effusion

After thoracentesis and pleura biopsy, pleural effusions were collected for further analysis. Bloody effusions (due to thoracic trauma or surgery) and effusions associated with air in the pleural space were not found in this study. Bilateral effusions were seen in 7 patients. Four cases had intrapulmonary involvement and intrapulmonary lesions presented as consolidation or infiltration. Bronchoscopy and transbroncial lung biopsy (TBLB) were performed in these patients, but eosinophilic infiltration was not found, and there was no evidence of tuberculosis or malignancy. Furthermore, two cases developed pericardial effusion as detected by chest CT (Fig. 1a and b).

### Eosinophils and other pleural parameters in pleural fluid

All pleural effusions were characterized as exudates according to Light’s criteria (effusion/serum protein ratio > 0.5, effusion/serum LDH ratio > 0.6), and EPE was detected in all patients. Eosinophilic count ranged from 12.4 to 50.5% with a median of 22.4%. The mean concentrations of pleural effusion lactate dehydrogenase (LDH), adenosine deaminase (ADA), protein, CEA and BNP were 246.0 U/L (range, 89.8–421.9 U/L), 13.8 U/L (range, 1.8–24.0 U/L), 42.6 g/dl (range, 32.8–52.6 g/dl) and 2.17 mg/mL (range, 0.46–4.31 mg/mL), and 1217.58 (range, 35.24–432.2 mg/mL), respectively (Table 3). Pleural effusion tuberculosis-DNA (TB-DNA), acid-fast bacilli smears and pleural effusion culture for fungi or bacteria were negative. Eosinophilic infiltration, lymphocyte infiltration, granulocytic infiltration and noncaseating granulomas were found in the pleural samples, but no evidence of either tuberculosis or malignancy was found in any of these patients.

**Table 3:** Pleura effusion characteristics of 11 patients with IEPE

No	Eos (%)	CEA (ng/mL)	ADA (U/L)	LDH (U/L)	Proteins (g/dl)	BNP (pg/mL)	TB-DNA	AFB smears	Culture of effusion
1	31	1.89	8.1	265	51.8	96.13	N	N	N
2	20	1.53	7.9	338.5	52.6	47.2	N	N	N
3	22.5	0.46	4	209	49.7	35.24	N	N	N
4	50.5	1.08	23.0	421.9	48.3	394.60	N	N	N
5	14.3	2.01	1.8	89.8	37.5	93.35	N	N	N
6	21	1.48	6.7	221.0	43.2	87.46	N	N	N
7	19	1.67	2.8	189.4	39.8	432.2	N	N	N
8	15.2	4.31	24.0	201.2	35.6	412	N	N	N
9	21.2	3.34	20.0	234.4	43.2	231.1	N	N	N
10	18.9	2.67	17	321.4	34.2	243.1	N	N	N
11	12.4	3.4	19.9	214.5	32.8	321.0	N	N	N

*Abbreviations: IEPE* idiopathic eosinophilic pleural effusion, *Eos* eosinophils, *CEA* carcinoembryonic antigen, *ADA* adenosine deaminase, *LDH* lactate dehydrogenase, *BNP* brain natriuretic peptide, *N* negative, *TB-DNA* tuberculosisDNA, *AFB* Acid-fast bacilli

Moreover, comprehensive haematological detection was performed in cases 2 and 11. Smear and biopsy of bone marrow showed no evidence of hypereosinophilia or infiltration indicative of lymphoproliferative malignancy. The possibility of myeloproliferative hypereosinophilic syndrome was excluded by negative FIP1L1-PDGFRA and BCR-ABL gene transcriptions. In cases 3 and 10, PET/CT was used as a systemic search to determine if the lungs and pericardium were involved, in addition to PE.

### Exploratory treatment and follow-up

After the initial diagnosis of IEPE, patients were treated with glucocorticoid (initial prednisone dose: 1 mg/kg of body weight per day). If the glucocorticoid resolved symptoms and abnormal pleural pulmonary radiographic signs, consecutive reduction of 10 mg per month was made. Physical examination with chest radiography, ultrasound and/or CT were followed up after the use of glucocorticoid.

**Fig. 2:**
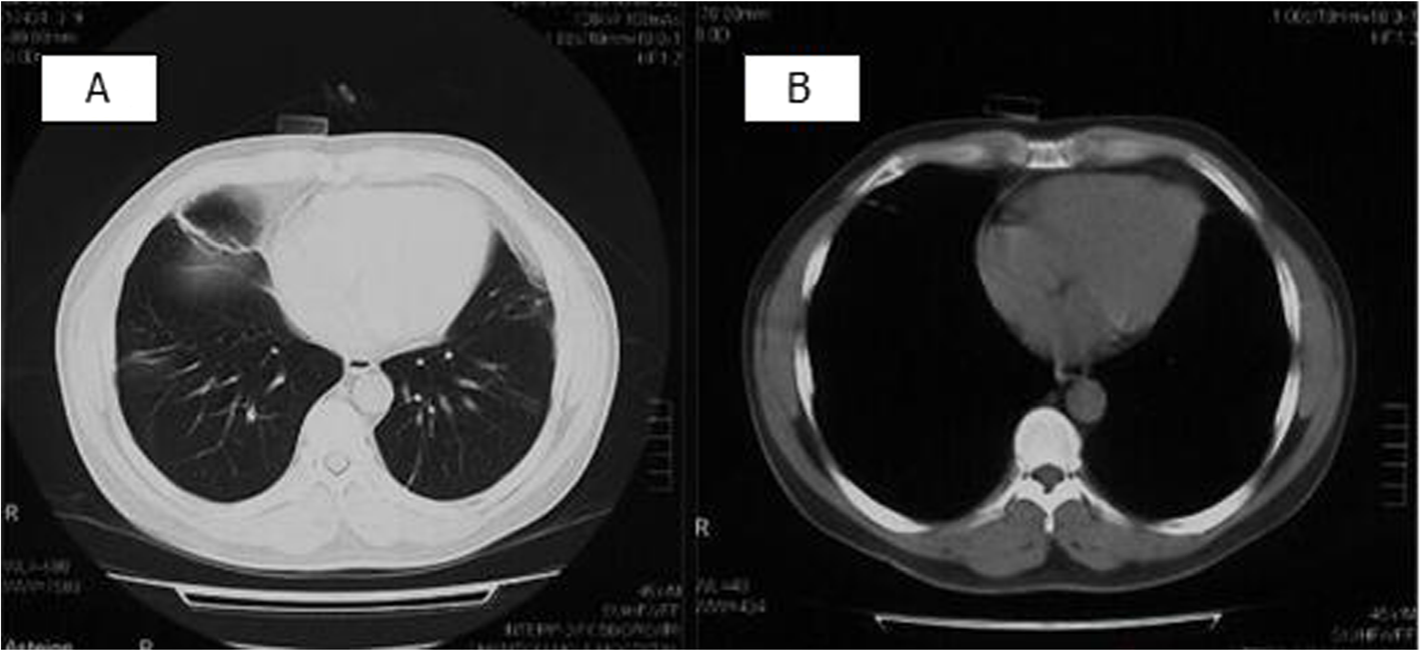
Follow-up chest CT scan of case 3. Total regression of PE, consolidation in the lower right lung and pericardial effusion with no recurrences

The median follow-up was 14.4 months (range, 8–16 months). All the patients showed total regression of the pleural effusion, without re-occurrence. These patients remained stable during follow-up and did not receive any additional therapy. Figure 2 shows the follow-up chest CT of a patient (case 3).

### Preliminary diagnostic procedure of IEPE

**Fig. 3:**
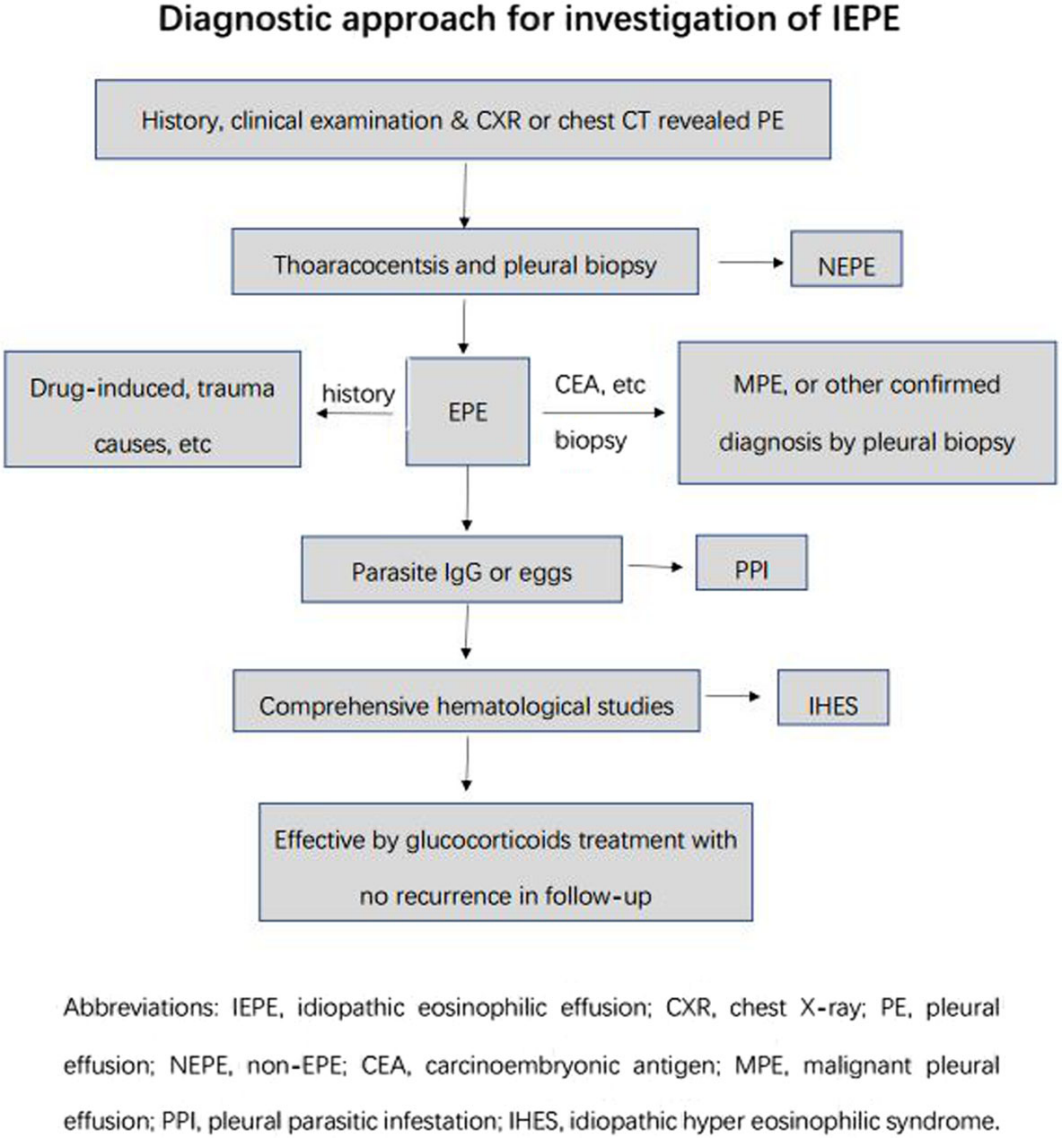
Schematic diagram of diagnostic procedure of IEPE

IEPE is a diagnosis of exclusion. Complete medical and surgical histories should be obtained from patients with EPE of unknown aetiology. It is recommended the patients should be followed up after receiving glucocorticoid, until the effusion resolves or a known cause becomes apparent. A preliminary diagnostic procedure of IEPE was developed and is shown in Fig. 3.

## Discussion

EPEs account for 5 to 16% of the exudative pleural effusions, and IEPE is an important cause of EPEs which can almost always be treated medically [7, 8]. However, well-documented cases are limited [12–16]. In this study, we tried to analyse the clinical characteristics of IEPE and to clarify the diagnostic procedure.

Archontogeorgis K et al. [19] first investigated the diagnostic approach in 10 patients with IEPE, but the clinical characteristics of IEPE were not assessed. In this study, the clinical features of IEPE were described in 11 prospective cases. Shortness of breath is one of major symptoms of IEPE. Moreover, fever, productive cough, fatigue, lymphadenopathy, splenomegaly and ascites often exist [7, 8]. Most patients had bilateral pleural effusion [11–13, 15], but unilateral effusion was also evident [14]. We found all 11 patients developed respiratory symptoms which are similar to IEPE symptoms. Among these cases, 7 had bilateral effusion and 4 had lung involvement. Previous reports [12–16] showed the eosinophils were always significantly elevated, reaching up to 3.5 × 10^9^/L. Contrarily, in this study, the number of eosinophils were normal or slightly elevated. Due to lack of pathognomonic characteristics and laboratory tests, some patients were initially misdiagnosed.

Current investigations of pleural effusions emphasise the use of a diagnostic algorithm or recommends the use of a stepwise approach [20–23]. Thoracocentesis was performed to ascertain the nature of pleural effusion and to differentiate it from other conditions. Consistent with the results of a previous study [19], pleural effusions in the 11 patients were exudative according to Light’s criteria. Pleural CEA, ADA and LDH were nonspecific in these 11 cases. Further prospective studies with larger sample sizes are needed to evaluate the diagnostic value of such effusion parameters.

A meta-analysis concluded that the most common cause of EPEs is malignancy (26%) [7]. Therefore, malignancy must be excluded as part of the diagnostic process for IEPE. CEA, a tumour marker, plays a role in MPE differentiation. Pleural CEA is often positive in suspected patients with malignancy [24, 25]. However, in our study, CEA were at normal level (< 5 mg/mL) in both the serum and PE. Archontogeorgis K et al. [19] emphasised that pleuroscopy is mandatory in diagnosing IEPE. Pleura biopsies seem to be mandatory when malignancy is excluded. It has been reported that if enough pleura biopsies are obtained, then the sensitivity and accuracy will increase to 88.6 and 93.8%, respectively [17], which would be comparable to the sensitivity and accuracy of a thoracoscopic examination [26]. In our study, pleural samples were collected by using combined ultrasound-guided cutting needle biopsy and standard pleural biopsy, without thoracoscopic assessment. Eosinophilic infiltration was found in 6 cases. However, there were no evidence of tuberculosis or malignancy in these patients.

Except for malignancy, the causes of EPE are varied and complicated, including parapneumonic effusions, pleural air/blood, tuberculosis, transudate, and collagen vascular disease [7, 8]. Therefore, a thorough work-up is essential in order to rule out known and obvious causes of EPE. In this study, relevant medications, autoimmune disease and chest trauma were not identified in any of the cases. Findings from a previous study [27] by the same authors confirmed that in patients with unexplained pleural effusion, parasite-specific IgG antibody detection had to be done when pleural fluid testing showed EPE. Physicians should consider a diagnosis of PPI when parasite-specific IgG antibody is positive. Based on this, we excluded PPI diagnosis.

Hypereosinophilic syndrome (HES) was redefined in 2010 as more than 1500/mm^3^ eosinophils without a discernible secondary cause (eg, HIV infection, parasite or worm infection, allergic diseases, drug allergies, and nonhematologic malignancies) [27]. Idiopathic HES (IHES) sometimes presents with EPE [28], but the causality of IHES and EPE has not been reached. Although the absolute eosinophil count was 2.16 × 10^9^/L in case 2, IHES diagnosis was excluded after comprehensive haematological determinations. Echocardiography displayed pericardial effusion in a case, while lung involvement was shown in 4 cases. Hence, PET/CT or transbronchial lung biopsy may be useful for verifying the diagnosis of chronic eosinophilic pneumonia in cases with lung involvement. When EPE has no apparent aetiology, the diagnosis of IEPE should be considered.

This study had limitations. This was a single-center experience with a small number of patients and therefore, the characteristics of IEPE were not able to be well-defined. A multicentre, prospective study, with a larger sample size, is needed to validate the findings of this study.

## Conclusions

IEPE is a diagnosis of exclusion. Complete medical, surgical, and drug-related histories should be obtained from patients with EPE of an unknown aetiology. A thorough work-up is essential. Furthermore, follow-up of the patient after the use of glucocorticoid until the effusion resolves is recommended.

## Data Availability

The datasets supporting the conclusions of this article are included within the article and its figures and tables. Additional data may be available from the corresponding author upon reasonable request.

## Abbreviations

EPE: Eosinophilic pleural effusion
IEPE: Idiopathic EPE
PE: Pleural effusion
HRCT: High-resolution computed tomography
MPE: Malignant PE
TPE: Tubercular PE
PPE: Parapneumonic PE
PPI: Pleural parasitic infestation
CHF: Chronic heart failure
PBC: Peripheral blood cell
IGRAs: Interferon-γ release assays
CEA: Carcinoembryonic antigen
BNP: Brain natriuretic peptide
TBLB: Bronchoscopy and transbroncial lung biopsy
LDH: Lactate dehydrogenase
ADA: Adenosine deaminase
TB-DNA: Tuberculosis-DNA
PET/CT: Positron emission tomography/computed tomography
HES: Hypereosinophilic syndrome
IHES: Idiopathic HES
